# Depression in Older Adults: Linking Daily Living Impairment and Socioeconomic Status

**DOI:** 10.1093/geroni/igaf122.2703

**Published:** 2025-12-31

**Authors:** Zeynep Abul, Sakeena Raza, Lynn McNicoll

**Affiliations:** Northeastern University, Boston, Massachusetts, United States; The Warren Alpert Medical School of Brown University, Providence, Rhode Island, United States; The Warren Alpert Medical School of Brown University, Providence, Rhode Island, United States

## Abstract

Depression is a significant public health problem among older adults. We aimed to investigate the association between depression, socioeconomic status, and activities of daily living (ADLs) in Health and Retirement Study 2018 data.A logistic regression analysis was conducted (n = 8,437). The dependent variable was an answer to a question about feeling depressed in the past week. Independent variables included education, ADL difficulties, gender, marital status, age, race/ethnicity, and household income. The model controlled for Hispanic ethnicity and racial categories, with appropriate reference groups. Study population includes 55% female, mean age of 75, 76% white individuals. ADL difficulties were associated with increased odds of depression. Each additional ADL limitation significantly increased odds of depression, with coefficients rising from β = 0.87 (1 ADL) to β = 1.96 (5+ ADLs), (p < 0.001). Females had higher odds of depression than males (β = 0.23, p = 0.004). Marital status like separation (β = 0.99 p < 0.001) or divorce (β = 0.38, p = 0.002) significantly increased depression. Hispanic ethnicity was associated with higher odds of depression compared to non-Hispanics (β = 0.58, p < 0.001). Lower education levels are linked to higher depression rates. Odds decrease as education increases: β=-0.32 for high school, β=-0.47 for some college, and β=-0.81 for college and above. Individuals with higher income are less likely to report depression (β = - 0.40, p < 0.001). Older individuals are less likely to report depression (β = -0.012, p = 0.02).Depression in older adults is associated with functional, social, and demographic factors in a complex way.

